# Blood pressure management in stroke: comparative review of the 2025 AHA/ACC/AANP/ACPM/AGS/AMA/ASPC/NMA/PCNA/SGIM, 2024 ESC, 2023 ESH, and 2025 JSH guidelines

**DOI:** 10.1038/s41440-025-02517-0

**Published:** 2026-01-09

**Authors:** Masatoshi Koga

**Affiliations:** Department of Cerebrovascular Medicine, National Cerebral and Cardiovascular Center, Osaka, Japan

**Keywords:** Ischemic stroke, Intracerebral hemorrhage, Acute stroke, History of stroke

## Abstract

Hypertension is the primary modifiable risk factor for both ischemic stroke and intracerebral hemorrhage (ICH), yet recommendations for blood pressure (BP) management vary across contemporary guidelines. This narrative review compares BP targets and therapeutic strategies in the 2025 American Heart Association (AHA), 2024 European Society of Cardiology (ESC), 2023 European Society of Hypertension (ESH), and 2025 Japanese Society of Hypertension (JSH) guidelines, with emphasis on acute and chronic phases of ischemic stroke and ICH. In acute ischemic stroke without reperfusion therapy, all four guidelines discourage routine BP lowering unless systolic BP (SBP) is ≥220 mmHg or diastolic BP ≥ 120 (110) mmHg, and then recommend only modest reductions of about 15% within 24 hours. For patients receiving IV thrombolysis or mechanical thrombectomy, the guidelines converge on pre-treatment BP<185/110 mmHg and maintenance <180/105 mmHg during the first 24 hours, with JSH specifying micro-infusion calcium channel blockers as preferred agents. In chronic ischemic stroke, AHA, ESH, and JSH generally endorse BP<130/80 mmHg, whereas ESC prioritizes an SBP range of 120-9 mmHg. For acute ICH, all guidelines support rapid but carefully titrated SBP reduction toward approximately 140 mmHg, while emphasizing avoidance of overshoot, large variability, and excessive early declines, particularly when baseline SBP exceeds 220 mmHg in the AHA and ESC guidelines. Long-term after ICH, targets of <130/80 mmHg are widely recommended. Thiazide diuretics, ACE inhibitors, and angiotensin receptor blockers remain foundational for secondary prevention, with calcium channel blockers central to acute parenteral therapy and β-blockers reserved for specific indications. Despite regional nuances, the guidelines converge on conservative acute management in ischemic stroke, proactive early lowering in ICH, and intensive long-term BP control as the global benchmark for secondary cerebrovascular prevention.

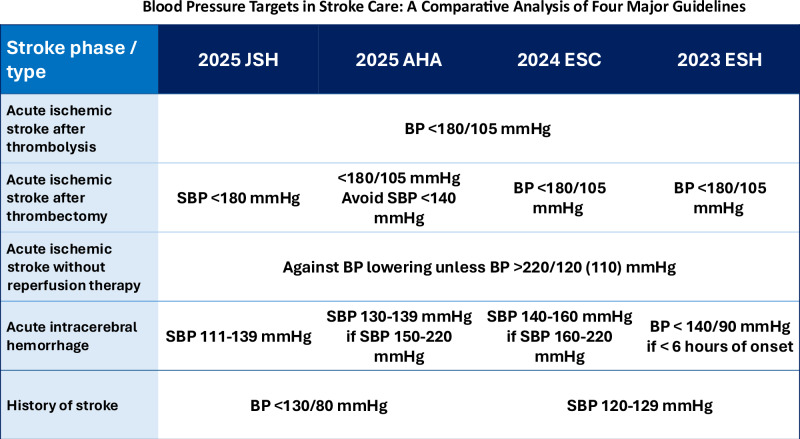

## Introduction

Blood pressure (BP) control remains a cornerstone in the acute and chronic management of cerebrovascular diseases. Hypertension is the single most important risk factor for both ischemic and hemorrhagic strokes, influencing not only stroke incidence but also outcomes, recurrence and cardiovascular risks. Clinical practice guidelines differ somewhat across regions, reflecting varying interpretations of trial evidence and local practice environments. This review compares the most recent four major guidelines—the 2025 AHA, 2024 ESC, 2023 ESH, and 2025 JSH hypertension guidelines—as summarized in Table 1 with emphasis on BP management in acute and chronic phases of ischemic stroke and intracerebral hemorrhage.

**Table 1:** Comparison of BP management in acute and chronic strokes among four major guidelines

	Conditions to treat		BP management
**Acute ischemic stroke candidates for thrombolytic therapy within 4.5 h after onset**			
2025 JSH 2025 AHA 2024 ESC	SBP ≥ 185 mmHg or DBP ≥ 110 mmHg		• BP < 185/110 mmHg prior to thrombolysis • BP < 180/105 mmHg for 24 h after thrombolysis
2023 ESH	N/A		• BP < 180/105 mmHg for 24 h after thrombolysis
**Acute ischemic stroke treated with mechanical thrombectomy within 24 h after onset**			
2025 JSH	SBP ≥ 180 mmHg		• SBP < 180 mmHg after thrombectomy
2025 AHA	SBP ≥ 180 mmHg DBP ≥ 105 mmHg		• BP < 180/105 mmHg for 24 h after thrombectomy • Avoid lowering SBP < 140 mmHg within the first 24-72 h
2024 ESC	SBP ≥ 185 mmHg or DBP ≥ 110 mmHg		• BP < 180/105 mmHg prior to and for 24 h after thrombectomy
2023 ESH	N/A		• BP < 180/105 mmHg for 24 h after thrombectomy
**Acute ischemic stroke without reperfusion therapy**			
2025 JSH 2025 AHA 2023 ESH	SBP > 220 mmHg or DBP > 120 mmHg		• By approximately 15% of the pretreatment BP value for first 24 h of onset (no description regarding the duration in the 2025 JSH)
2024 ESC	SBP > 220 mmHg or DBP > 110 mmHg		
**Acute intracerebral hemorrhage**			
2025 JSH	SBP ≥ 140 mmHg		• SBP > 110 and <140 mmHg • Avoid acute SBP reduction > 90 mmHg from the initial levels
2025 AHA	SBP between 150 and 220 mmHg		• SBP ≥ 130 and <140 mm Hg for at least 7 days • Stop medications if SBP < 130 mmHg
	SBP > 220 mmHg		• SBP between 160 and 180 mmHg • Avoid SBP < 130 mmHg
2024 ESC	SBP between 160 and 220		• SBP between 140 and 160 mmHg within 6 h of symptom onset
	SBP ≥ 220 mmHg		• Avoid acute SBP reduction >70 mmHg within 1 h of commencing treatment
2023 ESH	<6 h of symptom onset		• BP < 140/90 mmHg
	>6 h	SBP ≥ 220 mmHg	• SBP < 180 mmHg
		SBP < 220 mmHg	• Slow and moderate BP reductions
**History of ischemic stroke or intracerebral hemorrhage**			
2025 JSH 2025 AHA	SBP ≥ 130 mmHg or DBP > 80 mmHg		• BP < 130/80 mmHg
2024 ESC	SBP ≥ 130/80 mmHg		• SBP between 120 and 129 mmHg
2023 ESH	SBP ≥ 140 mmHg		• Initial BP target <140/80 mmHg, then BP < 130/80 mmHg, if tolerated • Avoid SBP < 120 mmHg

### Ischemic stroke

#### Acute ischemic stroke without reperfusion therapy

Excessive early BP lowering may worsen outcomes by reducing perfusion in the ischemic penumbra, as shown in ENCHANTED [1] and CATIS [2], which did not demonstrate functional benefit from early intensive BP lowering. For patients not undergoing reperfusion therapy, the 2025 AHA, 2024 ESC, 2023 ESH, and 2025 JSH uniformly recommend withholding antihypertensive treatment unless SBP ≥ 220 mmHg or DBP ≥ 120 (110) mmHg. If BP lowering is necessary, a modest reduction (≈15% in the first 24 hours) is advised.

### Acute ischemic stroke with thrombolysis or thrombectomy

All guidelines are highly consistent regarding BP thresholds for patients undergoing reperfusion therapies. The 2025 AHA recommends lowering BP<185/110 mmHg prior to intravenous thrombolysis and maintaining <180/105 mmHg for the first 24 hours. The 2024 ESC and 2023 ESH guidelines mirror these thresholds. The JSH 2025 provides similar recommendations, with specific mention of intravenous micro-drip of calcium channel blockers (nicardipine, diltiazem) as first-line options.

### History of ischemic stroke

To prevent secondary stroke, the 2025 AHA and JSH recommends lowering BP<130/80 mmHg. The 2025 JSH noted to avoid excessive lowering in patients with bilateral carotid stenosis (e.g., greater than 70% diameter stenosis) or major artery occlusion. The 2024 ESC recommend targeting systolic BP of 120-9 mmHg. The 2023 ESH stated that the goal BP should be below 130/80 mmHg, whenever possible and under clinical control [3]. It also noted that SBP values<120 mmHg should be avoided. SPS3 showed reduced ICH risk with intensive BP control (<130 mmHg) in lacunar stroke patients [4]. RESPECT also demonstrated reduced ICH with SBP<120 mmHg in patients with history of stroke [5].

### Intracerebral hemorrhage

#### Acute intracerebral hemorrhage

INTERACT2 demonstrated the trend of improved functional outcomes with intensive BP lowering (<140 mmHg) compared to <180 mmHg [6]. ATACH2 showed no significant difference between targeting SBP<140 mmHg versus 140 to <180 mmHg but found more renal adverse events with overly aggressive lowering [7].

The 2025 AHA recommends immediate lowering SBP to 130 to <140 mmHg for at least 7 days after onset in patients with SBP between 150 and 220 mmHg. For patients with SBP > 220 mmHg, SBP should not be lowered below 130 mmHg to reduce adverse events [7], and cautious, modest SBP reduction in the range of 160–80 mmHg is suggested. Though evidence is moderate and nonrandomized, it also recommends careful titration to ensure smooth, nonlabile, and sustained control of BP, avoiding peaks and large variability in SBP. The 2023 ESH advises rapid reduction to <140/90 mmHg within 6 hours of onset for early presenters, but in patients with SBP > 220 mmHg, careful lowering to <180 mmHg is acceptable for late presenters after 6 hours of onset, and slow and moderate BP reductions are preferable over intensive BP reductions to <140/90 mmHg. The 2024 ESC recommends that immediate BP lowering (within 6 h of symptom onset) should be considered to a systolic BP target 140-60 mmHg (typical achieved range in intensive trials) to prevent hematoma expansion and improve functional outcome. It also advises that, for those with systolic BP > 220 mmHg, acute reduction in systolic BP > 70 mmHg from initial levels within 1 h of commencing treatment is not recommended. The 2025 JSH recommends prompt lowering to <140 mmHg, but maintaining SBP > 110 mmHg and avoiding SBP drops >90 mmHg to prevent renal dysfunction.

### History of intracerebral hemorrhage

The PROGRESS trial confirmed the benefit of BP lowering (mean SBP reduction ≈9 mmHg) in reducing recurrent stroke (both ischemic and hemorrhagic) [8]. The 2025 AHA recommends office BP goal of <130/80 mmHg and the 2024 ESC and the 2023 ESH recommends a SBP target between 120 and 129 mmHg in patients with BP ≥ 130/80 mmHg. JSH 2025 recommends BP control <130/80 mmHg based on our systematic review and meta-analysis [9]. It also suggests that BP control <120/80 mmHg may be considered for patients at high risk of recurrent ICH (e.g., cerebral microbleeds, use of antithrombotics, and advanced age).

### Pharmacological considerations

Although the 2025 AHA have no specific recommendation in acute phase stroke, it stated that treatment with a thiazide-type diuretic, angiotensin-converting enzyme (ACE) inhibitors, or angiotensin II receptor blockers (ARBs) are recommended for lowering BP and reducing recurrent stroke and ICH risk in patients with hypertension who have experienced an ischemic stroke, or ICH. Although calcium channel blockers (CCBs) are recommended for the treatment of hypertension, and CCBs can effectively and safely lower BP in patients with a history of ischemic stroke or ICH based on our clinical experience, there are limited data on their efficacy for secondary stroke prevention.

There is no specific recommendation in the 2024 ESC. The 2023 ESH noted that prevention of stroke has been observed in large RCTs using different drug regimens. β blocker (BBs) are less effective for stroke prevention than the other major classes of antihypertensive agents and not considered as the preferred drugs, but BBs can be used in combination treatment, considering their specific indications and comorbidities.

The 2025 JSH described pharmacological therapy in acute and chronic stroke as separately. In acute stroke, nicardipine, diltiazem, nitroglycerin or nitroprusside (nitrate drugs) as a micro-dose drip intravenous infusion are recommended. Importantly, sublingual nifedipine capsules should not be used due to the risk of causing a sudden decrease in BP. Intravenous antihypertensive therapy should be switched to oral therapy as soon as feasible. In both acute and chronic stage, oral antihypertensive drugs include CCBs, ARBs, ACE inhibitors, and diuretics.

## Conclusions

Despite minor differences, the guidelines converge on key messages: in acute ischemic stroke, there are thresholds around reperfusion otherwise conservative unless ≥220/120 mmHg. In acute intracerebral hemorrhage, systolic BP should be lowered rapidly to ≈140 mmHg and avoid overshoot and variability. In chronic stroke, BP control <130/80 mmHg is the global benchmark.
